# Experimental Study on the Optimization of Coal-Based Solid Waste Filling Slurry Ratio Based on the Response Surface Method

**DOI:** 10.3390/ma15155318

**Published:** 2022-08-02

**Authors:** Zhen Wei, Ke Yang, Xiang He, Jiqiang Zhang, Guangcheng Hu

**Affiliations:** 1 State Key Laboratory of Mining Response and Disaster Prevention and Control in Deep Coal Mines, Anhui University of Science and Technology, Huainan 232001, China; sdweizhen0302@163.com (Z.W.); austzhangjq@163.com (J.Z.); 2 Institute of Energy, Hefei Comprehensive National Science Center, Hefei 230031, China; keyang2003@163.com; 3 Ningxia Hongdunzi Coal Industry, Yinchuan 753000, China; xlchi@aust.edu.cn

**Keywords:** coal-based solid waste, response surface methodology, interaction, regression model, multi-objective optimization

## Abstract

The large production and low comprehensive utilization rate of solid waste from coal power base affects the efficient and coordinated development of regional resources and the ecological environment. In order to promote utilization of solid waste from coal power base, coal gangue, fly ash, and gasification slag are mixed as raw materials to prepare filling materials, and a study on the evolution law of the mechanical properties of coal-based solid waste filling body is systematically carried out. After clarifying the physical and chemical properties of the filling materials, the Box–Behnken experimental design method was used to study the effects of slurry mass fraction, coal gangue, fly ash, and gasification slag on the strength of the filling body based on the response surface-satisfaction function coupling theory. Furthermore, a multivariate nonlinear regression model was constructed for the strength of the filling body at different maintenance ages. Based on the analysis of variance (ANOVA) and the response surface function, the impact mechanism of influencing factors and their interaction on the strength of filler were revealed. The results show that the strength of the filler is affected by single factors and interactions between factors. The interaction of slurry mass fraction and gangue dosing has a significant effect on the strength of the filler in the early stage; the interaction of fly ash and gangue dosing has a significant effect on the strength of the filler in the middle stage; the interaction of slurry mass fraction and gasification slag dosing has a significant effect on the strength of the filler in the final stage. The mixed filling materials significantly affect the strength of the filler as the maintenance time is extended. The mixed filling materials are extensively interlaced with the hydration products, calcium alumina, and calcium silicate hydrate (C-S-H) gel, forming a stable three-dimensional spatial support system as the maintenance time increases. The best ratio to meet the requirements of mine filling slurry pipeline transportation and filling body strength was selected using the regression model and the proposed economic function of filling material.

## 1. Introduction

In China, long-term, large-scale, and high-intensity coal mining has caused ecological damage, and a large amount of coal-based solid waste has accumulated with a low comprehensive utilization rate. With the rapid development of the economy, the demand for coal resources is increasing, and some mining areas are about to face three different mining situations, including below water bodies, below buildings, and below railroads [1,2]. Filling coal mining is a type of technology that avoids the pollution of solid waste and prevents or controls deep mine disaster efficiently, eventually realizing the green mining of coal resources [3,4,5]. While ensuring safe pumping of filling slurry, the filling body slurry ratio directly affects filling costs and mining disaster prevention. Therefore, it is necessary to deeply study the physical and chemical properties of filling materials and the strength evolution characteristics of filling body to promote a coordinated development of resources and the ecological environment of the coal power base, and therefore, realize the resource nation and effective utilization of bulk coal-based solid waste [6,7,8].

In recent years, much research on the physical and chemical properties of filling materials and filling slurry ratios has been conducted to realize the goal of building green mines and developing the green mining industry. Fall et al. established the satisfaction function of the filling body to provide a new method for optimizing material filling ratios [9]. Fu et al. obtained the filling body’s maximum strength when the waste rock to wind sand ratio was 7:3 [10]. In order to determine the optimal ratio of mixed aggregate, Gao et al. optimized the mixed filler ratio based on response surface theory and established the filler strength model [11]. Yang et al. optimized the mixed filler ratio of all-tailed sand-bar-ground sand to overcome the shortage of filler material in the Jinchuan mine [12]. Hou et al. designed the strength test of cemented filler using the RSM-BBD method and established a regression model of filler strength to optimize the filler material ratio [13]. Hu et al. analyzed the influence of various factors on the strength of the filler and proposed the optimization method of mixed filler ratio based on fractal theory [14]. Liu et al. defined cement, lime, and gypsum dosing as independent variables and filling body compressive strength as dependent variables, respectively, to explore the effect of factor interaction on filling body strength [15]. Qi et al. combined an enhanced regression tree with a grain swarm optimization algorithm to propose a filling body strength prediction model [16]. Based on the response surface-satisfaction function theory, Wu et al. constructed an evaluation index model for filling performance and investigated the functional relationship between influencing factors and response values and the optimal filling ratio under multi-objective conditions [17]. Using the central composite test scheme of the response surface method, Yin et al. constructed a multivariate nonlinear regression model of filling strength and revealed the influence mechanism of single factor multi-factor interaction on filling strength [18]. Fu et al. conducted a sensitivity analysis of filler strength and found that the filler strength obeyed an exponential function with mass fraction and a linear function with the ash/sand ratio [19]. Meng et al. developed a low-cost, high-quality filling cementitious material using slag micronized powder and studied the hydration mechanism of the new filling cementitious material [20].

The above studies achieved significant results in the optimization of mine filling slurry but ignored the influence of the interaction between various factors on the strength of the filling body. In view of this, coal-based solid waste was used as raw material in a large coal power plant in this research, the influence law of single factors, such as slurry mass fraction, coal gangue, fly ash, and gasification slag admixture, and their interaction on the strength of filler at different maintenance ages was studied. Moreover, a multivariate nonlinear regression model of filler strength was established, and a reasonable ratio of coal-based solid waste cemented filler was determined using a multi-objective optimization algorithm. This research provides theoretical guidance for mine filler and slurry preparation in mining.

## 2. Response Surface Function Method Optimization Theory

### 2.1. Response Surface Theory

In response surface theory, the response surface function is optimized according to the constraints through a series of deterministic experiments with reasonably selected test points and iterative strategies. Then, the magnitude of the independent variable x is solved by constructing a polynomial with a clear expression form, such as the response surface function $y_{1}=f(x)$ of the mapping function y=f(x). A commonly used second-order response surface function considering the interaction between independent variables is expressed as:

(1) $$y_{1}=g(x_{1},x_{2},\cdots ,x_{n})={\sum_{i=1}^{n}A_{ii}x_{i}}^{2}+\sum_{i=1}^{n-1}\sum_{j=1}^{n}A_{ij}x_{i}y_{j}+\sum_{i=1}^{n}A_{i}x_{i}+A_{0}$$

where $y_{1}$ is the response quantity, $x_{i}$ is the independent variable, n is the number of independent variables, $A_{0},\;A_{i},\;A_{i}{}_{j},\;A_{i}{}_{i}$ (i,j=1,2,3,⋯,n) are coefficients to be determined by iterating the test sample points, and the total number is (n+1)(n+2)/2.

Assuming that m times of trials are conducted, Y is the vector of dependent variables, and ε is the vector of unavoidable random errors in the trial, then:

(2) Y=AX+ε

where Y = ${\left[y^{\left(1\right)},y^{\left(2\right)},y^{\left(1\right)}\cdots ,y^{\left(m\right)}\right]}^{T}$ and $\varepsilon ={\left[\varepsilon_{1},\varepsilon_{2}\cdots ,\varepsilon_{m}\right]}^{T}$.

$$X=\left[\begin{array}{cccccccccccccccc} 1 & x_{1} & x_{2} & \cdots & x_{n} & x_{1}{}^{2} & x_{2}{}^{2} & \cdots & x_{n}{}^{2} & x_{1}x_{2} & x_{1}x_{3} & \cdots & x_{1}x_{n} & x_{2}x_{3} & \cdots & x_{n-1}x_{n}\\ 1 & x_{1} & x_{2} & \cdots & x_{n} & x_{1}{}^{2} & x_{2}{}^{2} & \cdots & x_{n}{}^{2} & x_{1}x_{2} & x_{1}x_{3} & \cdots & x_{1}x_{n} & x_{2}x_{3} & \cdots & x_{n-1}x_{n}\\ \cdot & \cdot & \cdot & & \cdot & \cdot & \cdot & & \cdot & \cdot & \cdot & & \cdot & \cdot & & \cdot \\ 1 & x_{1} & x_{2} & \cdots & x_{n} & x_{1}{}^{2} & x_{2}{}^{2} & \cdots & x_{n}{}^{2} & x_{1}x_{2} & x_{1}x_{3} & \cdots & x_{1}x_{n} & x_{2}x_{3} & \cdots & x_{n-1}x_{n} \end{array}\right]$$

$$A={\left[A_{0},A_{1},A_{2},\cdots ,A_{n},A_{1}{}^{2},A_{2}{}^{2},\cdots ,A_{n}{}^{2},A_{1}A_{2},A_{1}A_{3},\cdots ,A_{1}A_{n},A_{2}A_{3},\cdots ,A_{n}{}_{-1}A_{n}\right]}^{T}$$

Using least squares estimation, it can be obtained that:

(3) $$X^{T}XA=X^{T}Y$$

Two sides of Equation (3) are multiplied by the inverse $X^{T}X$ to obtain:

(4) $$A=(X^{T}X{)}^{-1}X^{T}Y$$

Based on the experimental data, the values of the coefficients to be determined A are calculated from Equations (1)–(4), and the response surface function is obtained from Equation (1). The response surface method is based on the experimental design for fitting the response surface function and significance analysis of the independent variables, and the commonly used experimental design methods are central combination design and Box–Behnken design, etc.

### 2.2. Satisfaction Function Multi-Objective Optimization Method

Because Equation (1) can generally only be optimally solved for response quantities one by one, it cannot simultaneously optimize multiple response quantities as a whole, and the optimal solution of the independent variables cannot be solved. By constructing a single satisfaction function for response quantities, Derringer, G. C. et al. proposed a multi-objective optimization method for solving response quantities with set target values, achieving greater satisfaction when the response value was closer to the target value [21]. The influencing factors of this experiment were the content of coal gangue, the content of fly ash, the content of gasification slag, and the mass fraction.

## 3. Filling Material Proportioning Test

### 3.1. Physicochemical Properties of Filling Materials

**Figure 1:**
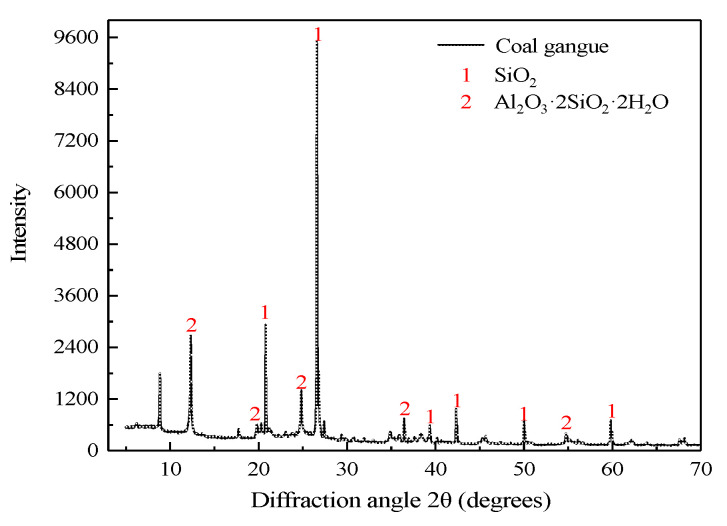
XRD diffraction qualitative analysis pattern of coal gangue.

The gangue used in the test was taken from the Renjiazhuang coal mine; it is not dewatered and dried but is crushed and sieved into coarse and fine aggregates. The grains with sizes of 0–5 mm and 5–10 mm were categorized as fine aggregate and coarse aggregate, respectively. The grain size composition of the gangue was measured by sieving using four-point sampling, and the specific grain size classification is shown in Table 1. A German D8-02 type X-ray (Bruker, Munich, Germany) diffraction instrument was used to determine the mineral composition of coal gangue, as shown in Figure 1. It can be seen that the main component of coal gangue is SiO_2_ and Al_2_O_3_; they are aluminium-silica-calcium material with low activity and are suitable for underground filling aggregates.

**Figure 2:**
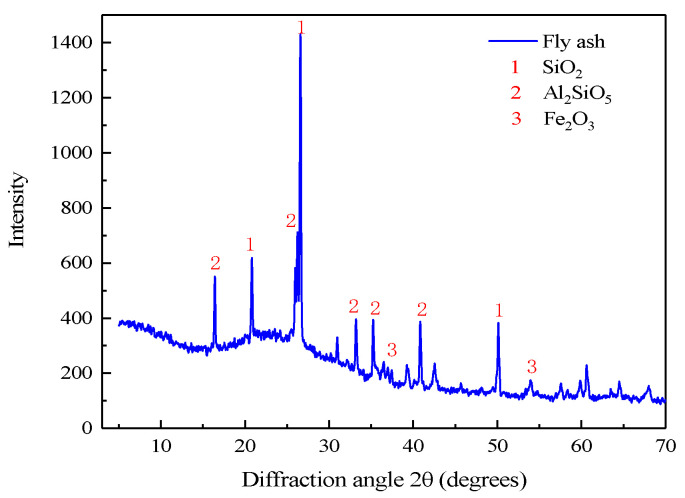
Qualitative analysis of fly ash XRD diffraction.

**Figure 3:**
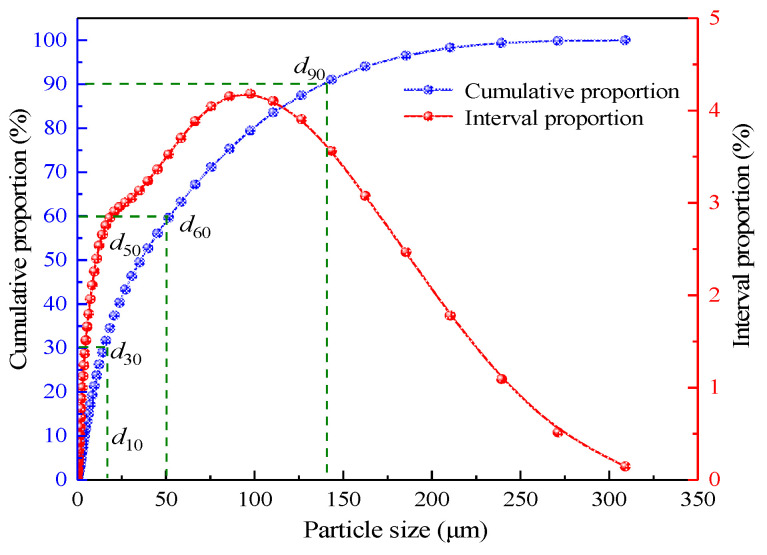
Fly ash particle size distribution curve.

In the test, the fly ash was taken from the coal-to-oil plant of Ningxia Coal Industry Co., its mineral composition and grain size distribution were, respectively, determined using a German D8-02 X-ray diffractometer and a British Mastersizer 3000 laser grain size analyzer, as shown in Figure 2 and Figure 3. It can be seen from Figure 2 that the main components of fly ash are SiO_2_ and Al_2_O_3_, which both are aluminium-silica-calcium substances with stable chemical properties and meet the requirements of underground filling materials. According to the grain size distribution curve, the grain size characteristic parameters of fly ash are determined to be $d_{10}$ = 4.58 μm, $d_{30}$ = 16.4 μm, $d_{50}$ = 35.3 μm, $d_{60}$ = 51.8 μm, $d_{90}$ = 144 μm, with the inhomogeneity coefficient $C_{u}$ = 11.31 and the curvature coefficient $C_{c}$ = 1.13, suggesting that the density is good. The surface of fly ash grains is smooth and regular in shape, making it conducive to slurry pipeline transportation and filling mine dewatering.

**Figure 4:**
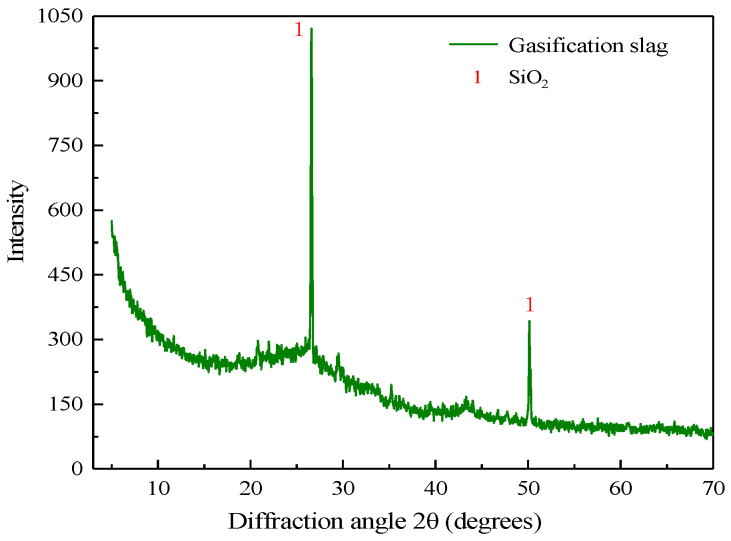
Qualitative XRD diffraction analysis profile of gasification slag.

**Figure 5:**
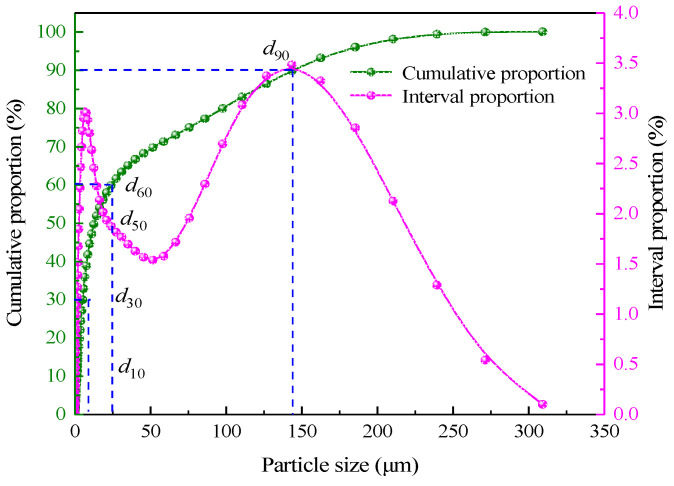
Particle size distribution curve of gasification slag.

The XRD diffraction pattern and grain size distribution of the gasification slag are shown in Figure 4 and Figure 5, and it can be seen that its main composition is SiO_2_, which is chemically stable. The grain size characteristic values of the gasification slag are $d_{10}$ = 1.45 μm, $d_{30}$ = 5.21 μm, $d_{50}$ = 12.7 μm, $d_{60}$ = 24.1 μm, and $d_{90}$ = 130 μm, and the inhomogeneity coefficient $C_{u}$ is 16.62 and the curvature coefficient $C_{c}$ is 0.77, indicating a non-uniform grain size distribution and high content of fine grains.

### 3.2. Proportioning Test Design

The parameters of filling body strength, slurry flow ability, and filling cost are critical indicators for evaluating the advantages and disadvantages of filling material proportioning. The mass fractions of coal gangue, fly ash, gasification slag, and mass fraction are used as the research objects expressed by $x_{1}$, $x_{2}$, $x_{3}$, and $x_{4}$, respectively. The compression strength of the filling body at 3, 7, and 28 d is represented by $y_{1}$, $y_{2}$, and $y_{3}$, respectively. Taking into account the filling slurry secretion rate, diffusivity and test cost, the levels of each factor were respectively classified as follows: 0.1, 0.2, and 0.3 for coal gangue; 0.65, 0.73, and 0.8 for fly ash; 0.1, 0.15, and 0.2 for gasification slag; and 72%, 77%, and 82% for the slurry mass fraction. Cement accounted for 10% of the total mass of solid waste in each group of tests. The response surface test design method has a small number of tests, which can reveal the influence of each test factor and their interaction on the strength of the filler according to the limited number of tests; the test factor levels are shown in Table 2.

Following the test program, coal gangue, fly ash, gasification slag, cement, and water were weighed and mixed for 5 min using a mortar mixer to ensure the uniformity of slurry and eliminate internal air bubbles, avoiding the impact on the strength of the test block. The mixed slurry was poured evenly into the standard triplex mould with the size of 70.7 mm × 70.7 mm × 70.7 mm. After the slurry filled in the mould and settled naturally, the mould was moved to a compacting table to vibrate and compact. Then, the mould was put into an HSB-40B constant temperature and humidity curing box with a curing temperature of 20 °C and a curing humidity of 90%; after 24 h, the specimens were demolded and cured for 3, 7, and 28 d. The uniaxial compressive strength of the filled body was tested using a WEW-600D hydraulic universal testing machine. The strengths of the filling bodies with different ratios were measured according to the test protocol, as shown in Table 3.

## 4. Experimental Results and Analysis

### 4.1. Response Surface Function Fitting

According to the strength test results of different ratios of the filler, the multivariate nonlinear regression fitting was performed using Design-Expert software, and the response surface functions of the uniaxial compressive strength of the filler specimens at 3, 7, and 28 d were obtained, as shown in Equations (5)–(7). The fitted correlation coefficients of the three response surface functions were 0.9892, 0.9650, and 0.9267, which were close to the ideal value of one, i.e., the response equation fits well.

(5) $$\begin{array}{l} y_{1}=0.0041x_{1}{}^{2}+0.0516x_{2}{}^{2}+0.0453x_{3}{}^{2}+0.3953x_{4}{}^{2}\\ -0.27x_{1}x_{2}+0.05x_{1}x_{3}+0.2325x_{1}x_{4}-0.07x_{2}x_{3}+0.0875x_{2}x_{4}-0.23x_{3}x_{4}\\ +0.0142x_{1}-0.708x_{2}+0.02x_{3}+0.5783x_{4}+0.976(R^{2}=0.9892) \end{array}$$

(6) $$\begin{array}{l} y_{2}=-0.0476x_{1}{}^{2}+0.0787x_{2}{}^{2}-0.0813x_{3}{}^{2}+0.3274x_{4}{}^{2}\\ -0.2475x_{1}x_{2}+0.14x_{1}x_{3}+0.2025x_{1}x_{4}+0.115x_{2}x_{3}+0.16x_{2}x_{4}-0.1775x_{3}x_{4}\\ -0.0917x_{1}-0.0658x_{2}-0.0125x_{3}+0.77x_{4}+1.45(R^{2}=0.9650) \end{array}$$

(7) $$\begin{array}{l} y_{3}=-0.1256x_{1}{}^{2}+0.1935x_{2}{}^{2}+0.0797x_{3}{}^{2}+0.4997x_{4}{}^{2}\\ -0.2725x_{1}x_{2}+0.0925x_{1}x_{3}+0.1725x_{1}x_{4}-0.0325x_{2}x_{3}+0.1125x_{2}x_{4}-0.42x_{3}x_{4}\\ +0.0042x_{1}-0.0792x_{2}+0.0933x_{3}+0.8783x_{4}+1.65(R^{2}=0.9267) \end{array}$$

**Figure 6:**
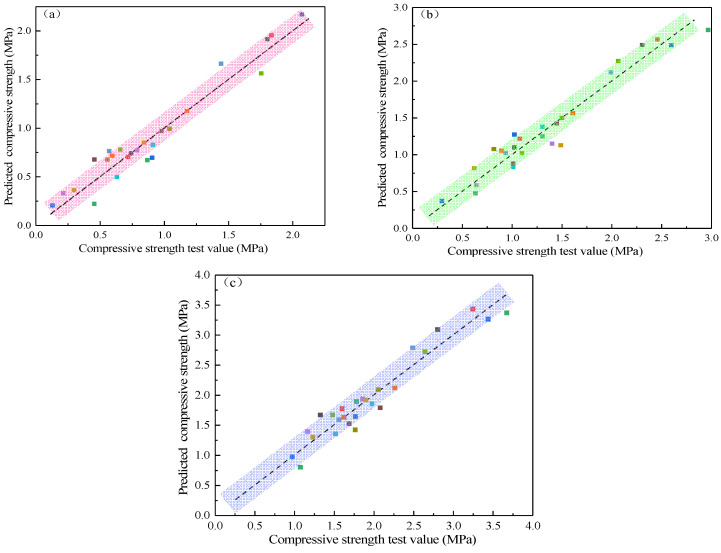
Comparison of experimental and predicted values of filler strength: (**a**) 3 day; (**b**) 7 day; (**c**) 28 day.

The function regression models of the response surface were subjected to the analysis of variance (ANOVA) to assess their accuracy, as shown in Table 4. It can be found that the P of all three models is less than 0.0001, indicating that the regression effect of the model is significant, credible and statistically significant. The significance of the model was determined according to the values of F and P; the larger the value of F and the smaller the value of P, the more significant the effect. The uniaxial compressive strength on the third day was ranked by the significance of the single factor as $x_{4}>x_{2}>x_{3}>x_{1}$, and by the interaction of factors as $x_{1}x_{2}>x_{1}x_{4}>x_{3}x_{4}>x_{2}x_{4}>x_{2}x_{3}>x_{1}x_{3}$. The significance of the influence of a single factor on the uniaxial compressive strength on the seventh day was ranked as $x_{4}>x_{3}>x_{2}>x_{1}$, and the significance of the interaction of various factors was ranked as $x_{1}x_{2}>x_{1}x_{4}>x_{3}x_{4}>x_{2}x_{4}>x_{1}x_{3}>x_{2}x_{3}$. The order of significance of single factor influence on uniaxial compressive strength on the 28th day was $x_{4}>x_{1}>x_{2}>x_{3}$, and the order of significance of the interaction of various factors was $x_{3}x_{4}>x_{1}x_{2}>x_{1}x_{4}>x_{2}x_{4}>x_{1}x_{3}>x_{2}x_{3}$. A comparison of the experimental and predicted values of the strength of the filler in different maintenance ages is shown in Figure 6. It can be seen that the scatter points are distributed around the straight line y=x, i.e., the experimental values are in good agreement with the predicted values, and the age strength model is valid.

### 4.2. Influence of Single Factors of Response Surface Parameters on the Strength of the Filled Body

**Figure 7:**
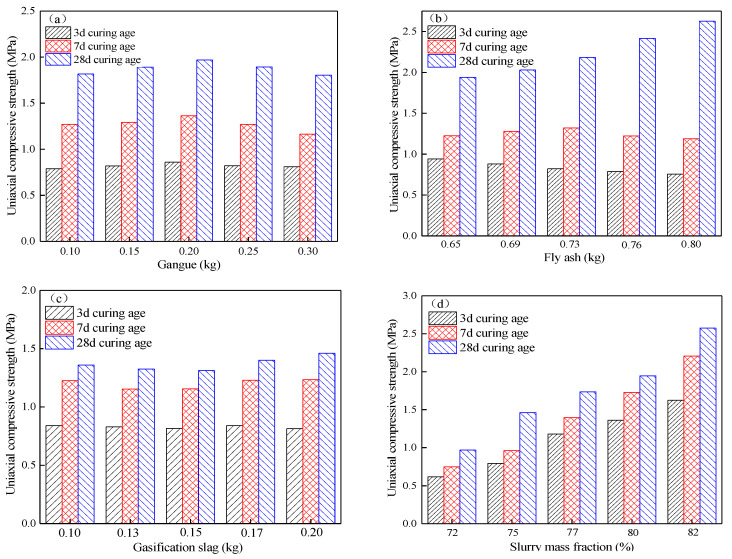
Effect of single factors on the strength of the filling body: (**a**) gangue; (**b**) fly ash; (**c**) gasification slag; (**d**) slurry mass fraction.

When fly ash is 0.73 kg, gasification slag is 0.15 kg, and the slurry mass fraction is 77%, the relationship between the amount of coal gangue doping and the compression strength of the cemented filling body at different maintenance ages is shown in Figure 7a. It can be seen that with an increase in the amount of gangue, the strength of the filler first increases and then decreases; this is because as the “skeleton” of the filler, the number of pores in the test block increases with an increase in the amount of gangue, resulting in a reduction in the strength of the filler; the strength of the filler reaches the maximum when the amount of gangue is 0.2 kg. Combined with Table 4, the regression model of compression strength of gangue admixture $x_{1}$ at each maintenance age has the smallest *F* value, suggesting that the influence of gangue admixture on the strength of the cemented filling body is less than the fly ash admixture and slurry mass fraction. Under the condition of constant fly ash dose, gasification slag dose, and slurry mass fraction, solid fillers have good packing compactness and stable space structure when the coal gangue dose is 0.2 kg, making the strength of cemented filler reach the maximum value.

When the coal gangue is 0.2 kg, the gasification slag is 0.15 kg, and the slurry mass fraction is 77%, the relationship between the fly ash admixture and the compressive strength of the cemented filler at each maintenance age is shown in Figure 7b. It can be seen that the strength of the filled body on the third day decreases with an increase in fly ash admixture, which is around 0.8 MPa. The strength of the filled body on the seventh day increases with an increase in fly ash admixture, and then decreases, and the compressive strength reaches the maximum value of 1.32 MPa at 0.73 kg of fly ash admixture. The strength of the filled body on the 28th day increases with an increase in fly ash admixture, mainly due to the hydration reaction of cement to produce Ca(OH)_2_ and the secondary reaction of fly ash, which further improves the compactness of the specimen. Therefore, fly ash admixture has a weakening effect on the filled body’s early strength and a significant increasing effect on the later strength.

When the gangue is 0.2 kg, fly ash is 0.73 kg, and the slurry mass fraction is 77%, the relationship between the dosing of gasification slag and the compressive strength of the cemented filler at each maintenance age is shown in Figure 7c. It can be seen that the strength of the filling body in the early stage does not change significantly with an increase in gasification slag, and the strength in the later stage first decreases, and then increases with the gasification slag, and the changes are within 5%; for example, the strength of the filling body at different maintenance ages changes slightly with an increase in gasification slag, which is consistent with the ANOVA results. The grain size of gasification slag is mainly concentrated in 0.4–4.75 mm, which is much larger than the grain size of fly ash, and there are insoluble residues in the gasification slag in the form of flocculent, making it difficult to form a perfect three-dimensional spatial support system for the filling body.

When the gangue is 0.2 kg, fly ash is 0.73 kg and gasification slag is 0.15 kg, the relationship between the slurry mass fraction and the compressive strength of the cemented filler at each curing age is shown in Figure 7d. It can be seen that the strength of the filled body at each curing age increases with the slurry mass fraction, and the higher the slurry mass fraction, the more significant the increase in the strength. When the mass fraction of slurry is 72–77%, the increasing rate of strength of the filled body increases with the mass fraction, and when the mass fraction of slurry is 77–82%, this increasing rate decreases, indicating that the mass fraction of slurry has more influence on the early strength of the filled body, which is consistent with the ANOVA results.

The mass fraction of the slurry determines the amount of water in the slurry. At the beginning of the hydration reaction, a large amount of columnar calcium alumina is generated inside the filler, accompanied by a small amount of calcium silicate gel. The hydration products fill the gaps between gangue grains and gasification slag grains, and the microstructure of the filler increases in compactness and adhesion [22]. As the mass fraction of slurry gradually increases, it becomes difficult for the amount of water in the test block to make all the cement hydration reactions, and the generation rate of hydration products slows down, resulting in the increasing rate of filling body strength decreasing with the mass fraction. In addition, the mass fraction can effectively increase the viscosity of the filling slurry, increase the settling resistance of the aggregate in the slurry, reduce water secretion and stratification of the aggregate, make the aggregate distribution more uniform, and thus improve the strength of the filling body.

### 4.3. Effect of Response Surface Parameter Interactions on the Strength of the Filler

The spatio-temporal distribution of the multi-component mixed filling materials significantly affects the strength of the filling body. The filling body strength is influenced by single factors and multi-factor interactions, and their relationships show nonlinearity. The interaction between slurry mass fraction and gangue in the mixed filling material has a significant effect on the strength of the filling body on the third day, *p* < 0.05; the interaction between fly ash and gangue has a significant effect on the strength of the filling body on the seventh day, *p* < 0.05; the interaction between slurry mass fraction and gasification slag has a significant effect on the strength of the filling body on the 28th day, *p* < 0.05.

**Figure 8:**
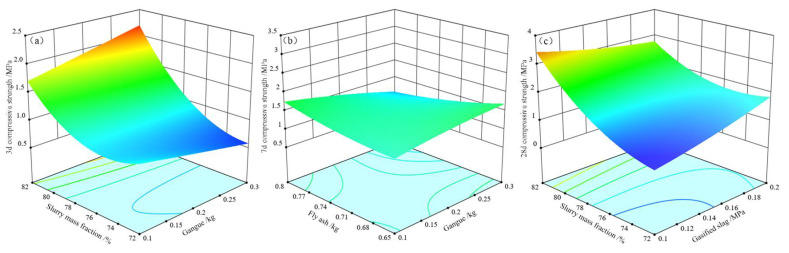
Effect of interaction of response surface factors on the strength of the filler: (**a**) The effect of interaction between slurry mass fraction and gangue dosing on the 3-day strength of the filler; (**b**) the effect of interaction between fly ash and gangue dosing on the 7-day strength of the filler; (**c**) the effect of interaction between slurry mass fraction and gasification slag dosing on the 28-day strength of the filler.

The effect of the interaction between the slurry mass fraction and gangue doping in the mixed filling on the strength of the filling body on the third day is shown in Figure 8a. It can be seen that the strength increases with an increase in slurry mass fraction, and first increases with the amount of gangue, and then decreases. When the mixture of gangue was 0.2 kg, as the slurry mass fraction changed from 72% to 82%, this strength was increased by 165%; when the mixture of gangue was 0.3 kg, as the slurry mass fraction changed from 72% to 82%, this strength was increased by 103%. It can be seen that the sensitivity of 3-day strength to the slurry mass fraction increases with the amount of gangue for the same aggregate ratio, and this strength increases significantly with the slurry mass fraction. In the early maintenance of the filler specimen, the cement hydration reaction did complete, resulting in a small amount of calcium silicate hydrate (C-S-H) gel which is difficult to fill the pores fully; at this time, the filler strength is mainly provided by the “skeleton”, and an increase in the slurry mass fraction is conducive to the formation of a more stable skeletal system of the filler. Therefore, increasing the slurry mass fraction by improving the aggregate ratio in a certain range effectively promotes the development of early filler strength.

The effect of the interaction between fly ash and gangue doping in the mixed filling material on the strength of the filling body on the seventh day is shown in Figure 8b. It can be seen that mixed filling material in the fly ash doping was low, and this strength increased with the amount of coal gangue. When the fly ash was 0.65 kg, as coal gangue changed from 0.1 kg to 0.3 kg, filling body strength was increased by about 27%. When mixed filling material in the coal gangue doping was high, this strength first increased, and then decreased with the amount of fly ash. When the coal gangue was 0.3 kg, as fly ash increased from 0.65 kg to 0.8 kg, the strength of the filling body was reduced by about 36%. The good aggregate ratio improves the stacking density of the mixture and forms a stable skeleton structure of the filler. In the middle stage of the maintenance of the filler specimen, the cement hydration reaction becomes more adequate, the gel is coherent into chains, and the generated calcium silicate hydrate (C-S-H) gel glues the skeleton structure into a whole, improving the strength of the filler. Therefore, the interaction between fly ash and gangue dosing has a more significant effect on the strength of the filler on the seventh day.

The effect of the interaction between slurry mass fraction and gasification slag dosing in the mixed filler on the strength of the filler on the 28th day is shown in Figure 8c. It can be seen that this strength increased with the slurry mass fraction, and the growth rate was fast when the dosing of gasification slag became higher, and the filler strength was increased by about 215%. When the gasification slag was 0.3 kg, as the slurry mass fraction increased from 72% to 82%, this strength showed a slight trend of first decreasing, and then increasing with the gasification residue, which was insoluble in water and often suspended in the filling slurry in flocculent form, resulting in uneven distribution of slurry aggregates and a reduction in strength of the filling body. The ratio of mixed filler is closely related to the slurry mass fraction, and various types of solid waste determine the performance of mixed filler. Under a reasonable aggregate ratio, increasing the slurry mass fraction reduces the occurrence of delamination and segregation [23,24]. Therefore, the interaction between slurry mass fraction and gasification slag dosing significantly affects the strength of the filler in the final stage.

## 5. Multi-Objective Optimization of Filling Slurry Ratio

Filling cost has a significant impact on the economic benefits of a mine under the premise of meeting the requirements of mine filling. Therefore, the strength of filling body on the 3rd day, 7th day, and 28th day is required to be greater than 1 MPa, 1.5 MPa, and 2 MPa, respectively. For per unit volume of filling slurry, the mass of coal gangue $M_{1}$, fly ash $M_{2}$, the mass of gasification slag $M_{3}$, and the mass of water $M_{4}$ can be calculated according to the amount of coal gangue blending ($x_{1}$), fly ash blending ($x_{2}$), and gasification slag blending ($x_{3}$). The price of cement is 300 yuan/t, coal gangue is 20 yuan/t, fly ash is 80 yuan/t, gasification slag is 120 yuan/t, and industrial water is 3.2 yuan/t. Considering the filling effect and economic cost, the lowest filling cost per unit volume is used as the optimization objective, and the age strength of the filling body is used as the constraint to establish the optimization solution model:

(8) $$f=30(M_{1}+M_{2}+M_{3})+20M_{1}+80M_{2}+120M_{3}+3.2M_{4}$$

(9) $$y_{1}\geq 1;\;y_{2}\geq 1.5;\;y_{3}\geq 2$$

(10) $$\frac{M_{1}}{2732}+\frac{M_{2}}{1583}+\frac{M_{3}}{2360}+\frac{M_{4}}{1000}+\frac{0.1(M_{1}+M_{2}+M_{3})}{1300}=1$$

(11) $$\frac{M_{1}+M_{2}+M_{3}+0.1(M_{1}+M_{2}+M_{3})}{M_{1}+M_{2}+M_{3}+M_{4}+0.1(M_{1}+M_{2}+M_{3})}=x_{4}$$

where f is the cost per volume of filling slurry, yuan/m^3^. Combining the equations from (5) to (11), the satisfying solution can be solved. To meet the expectation for the lowest filling cost, the optimal slurry ratio of $x_{1}$ = 0.22 kg, $x_{2}$ = 0.75 kg, $x_{3}$ = 0.14 kg, $x_{4}$ = 76% was selected, and the corresponding strength of the filling body on the third day was 1.22 MPa and the slurry collapse degree was 267 mm, which met the requirements of pipeline transportation of filling slurry and strength of filling body. Furthermore, by substituting the optimal filling material ratio into Formula (8), the filling cost coud be calculated as 115.62 yuan/m^3^.

## 6. Discussion

Based on the strength evolution characteristics of filling slurry at different ages, it can be seen that a large number of hydration products are produced in the early stage mainly in the form of fine filaments and flocs, which is a necessary condition for the early strength of cemented filling [25,26,27]. Filiform crystals cross-fill the grain pores, and a relatively complete network structure is formed, whose structure is loose, and the pore size is large, therefore, the early strength of the cemented backfill is not high. With the hydration reaction, the hydration products with flocculent structure develop into agglomerates, which are closely bonded with ettringite to fill the gap of aggregate grains, and a small amount of them cover the surface of aggregate grains. The three cooperate to form a dense and solid internal structure of the filler [28]. Mine backfill mining requires that the strength of the backfill body is greater than 1 MPa at 3 d of age, greater than 1.5 MPa at 7 d of age, and greater than 2 MPa at 28 d of age. Therefore, the samples that meet the strength requirements of filling and mining can be applied to a engineering site.

## 7. Conclusions

Multivariate nonlinear regression models of composite filler on the 3rd day, 7th day, and 28th day were constructed using response surface theory design, and the model correlation coefficients were 0.9892, 0.9650, and 0.9267, respectively. The difference between the experimental and predicted values of compressive strength of filler under different maintenance ages was less than 0.23 MPa. The significance test of the regression model was carried out, and indicated that the filler age strength model was valid, the prediction accuracy was high, and the fitting effect was good, which could provide a scientific test method with a reference value and guidance for site design.

ANOVA determined the significance of response surface parameters at different ages and explored the nonlinear influence law of single factors and their interaction on the strength of cemented filler with the help of the response surface model. The study results show that the interaction between slurry mass fraction and gangue dosing has a significant effect on the early strength of the filler, the interaction between fly ash and gangue dosing has a significant effect on the middle strength of the filler, and the interaction between slurry mass fraction and gasification slag dosing has a significant effect on the late strength of the filler.

The slurry economic function that considered the filling cost was established through multi-objective optimization. The optimal ratio of filling material was obtained under the constraint of filling body strength requirement at each age as 0.22 kg of coal gangue, 0.75 kg of fly ash, 0.14 kg of gasification slag, and 76% of slurry mass fraction, at which time the 3-day strength of filling body was 1.22 MPa and the slurry collapse degree was 267 mm. The filling slurry can meet the requirements of mine filling slurry pipeline transportation and filling body strength. At this time, the filling cost is 115.62 yuan/m^3^.

## Figures and Tables

**Table 1:** Gangue size classification.

Grain size (mm)	0–5	5–10	10–15	>15
Percentage (%)	24.8	32.7	28.3	14.2

**Table 2:** Test factor range settings.

Factors	Variables	Level		
Coal gangue	$x_{1}$	0.1 kg	0.2 kg	0.3 kg
Fly ash	$x_{2}$	0.65 kg	0.73 kg	0.8 kg
Gasification slag	$x_{3}$	0.1 kg	0.15 kg	0.2 kg
Mass fraction	$x_{4}$	72%	77%	82%

**Table 3:** Experimental factor range settings.

Number	$x_{1}\;(\mathrm{kg})$	$x_{2}\;(\mathrm{kg})$	$x_{3}\;(\mathrm{kg})$	$x_{4}\;(\%)$	$y_{1}\;(\mathrm{MPa})$	$y_{2}\;(\mathrm{MPa})$	$y_{3}\;(\mathrm{MPa})$
1	0.2	0.8	0.15	82	1.97	3.18	4.38
2	0.2	0.65	0.15	82	1.96	2.74	3.52
3	0.2	0.8	0.2	77	1.13	1.23	1.89
4	0.1	0.73	0.15	82	1.88	2.21	2.61
5	0.1	0.65	0.15	77	0.94	1.58	1.65
6	0.2	0.73	0.15	77	0.86	1.53	1.77
7	0.3	0.8	0.15	77	0.54	0.90	1.13
8	0.1	0.73	0.2	77	0.80	1.33	1.50
9	0.1	0.73	0.1	77	0.88	1.50	1.52
10	0.2	0.65	0.1	77	0.97	1.63	1.66
11	0.2	0.73	0.2	82	1.63	2.14	2.48
12	0.1	0.8	0.15	77	1.25	1.77	1.68
13	0.3	0.73	0.2	77	1.12	1.65	2.06
14	0.2	0.73	0.15	77	0.86	1.23	1.60
15	0.2	0.73	0.15	77	0.84	1.16	1.24
16	0.3	0.65	0.15	77	1.41	1.70	2.02
17	0.1	0.73	0.15	72	1.03	1.20	1.41
18	0.3	0.73	0.15	82	2.38	2.40	2.78
19	0.3	0.73	0.15	72	0.50	0.53	0.96
20	0.2	0.73	0.15	77	1.15	1.32	1.46
21	0.2	0.65	0.2	77	1.30	1.25	2.19
22	0.2	0.73	0.1	72	0.80	0.91	1.03
23	0.2	0.8	0.1	77	1.08	1.15	1.59
24	0.2	0.73	0.15	77	1.01	1.16	1.48
25	0.2	0.8	0.15	72	0.56	0.92	1.19
26	0.3	0.73	0.1	77	1.00	1.26	1.75
27	0.2	0.73	0.2	72	1.17	1.23	1.94
28	0.2	0.65	0.15	72	0.90	1.08	1.31
29	0.2	0.73	0.1	82	2.18	2.82	4.25

**Table 4:** Response surface regression model analysis of variance.

Variation Source	Square and			Mean Square			F-Value			*p*-Value		
	$y_{1}$	$y_{2}$	$y_{3}$	$y_{1}$	$y_{2}$	$y_{3}$	$y_{1}$	$y_{2}$	$y_{3}$	$y_{1}$	$y_{2}$	$y_{3}$
Models	5.91	8.94	12.72	0.4222	0.6387	0.9087	15.82	11.20	9.86	<0.0001	<0.0001	<0.0001
$x_{1}$	0.0024	0.1008	0.0002	0.0024	0.1008	0.0002	0.0903	1.77	0.0023	0.7683	0.2050	0.9627
$x_{2}$	0.0602	0.0520	0.0752	0.0602	0.0520	0.0752	2.26	0.9116	0.8163	0.1553	0.3559	0.3816
$x_{3}$	0.0048	0.0019	0.1045	0.0048	0.0019	0.1045	0.1799	0.0329	1.13	0.6779	0.8587	0.3048
$x_{4}$	4.01	7.11	9.26	4.01	7.11	9.26	150.43	124.71	100.48	<0.0001	<0.0001	<0.0001
$x_{1}x_{2}$	0.2916	0.2450	0.2970	0.2916	0.2450	0.2970	10.93	4.29	3.22	0.0052	0.0472	0.0942
$x_{1}x_{3}$	0.0100	0.0784	0.0342	0.0100	0.0784	0.0342	0.3748	1.37	0.3715	0.5502	0.2607	0.5520
$x_{1}x_{4}$	0.2162	0.1640	0.1190	0.2162	0.1640	0.1190	8.10	2.88	1.29	0.0129	0.1121	0.2748
$x_{2}x_{3}$	0.0196	0.0529	0.0042	0.0196	0.0529	0.0042	0.7346	0.9272	0.0459	0.4058	0.3519	0.8335
$x_{2}x_{4}$	0.0306	0.1024	0.0506	0.0306	0.1024	0.0506	1.15	1.79	0.5495	0.3021	0.2017	0.4708
$x_{3}x_{4}$	0.2116	0.1260	0.7056	0.2116	0.1260	0.7056	7.93	2.21	7.66	0.0137	0.1594	0.0151
$x_{1}{}^{2}$	0.0001	0.0147	0.1038	0.0001	0.0147	0.1038	0.0041	0.2574	1.13	0.9501	0.6198	0.3065
$x_{2}{}^{2}$	0.0173	0.0401	0.2429	0.0173	0.0401	0.2429	0.6469	0.7036	2.64	0.4347	0.4157	0.1268
$x_{3}{}^{4}$	0.0133	0.0429	0.0413	0.0133	0.0429	0.0413	0.4996	0.7521	0.4478	0.4913	0.4004	0.5143
$x_{4}{}^{2}$	1.01	0.6954	1.62	1.01	0.6954	1.62	38.00	12.19	17.58	<0.0001	0.0036	0.0009

## Data Availability

The data used in this research has been properly cited and reported in the main text.
